# Imide arylation with aryl(TMP)iodonium tosylates

**DOI:** 10.3762/bjoc.14.90

**Published:** 2018-05-11

**Authors:** Souradeep Basu, Alexander H Sandtorv, David R Stuart

**Affiliations:** 1 Department of Chemistry, Portland State University, Portland OR 97201, United States

**Keywords:** arylation, C–N coupling, diaryliodonium, hypercoordinate iodine, metal-free

## Abstract

Herein, we describe the synthesis of *N*-aryl phthalimides by metal-free coupling of potassium phthalimide with unsymmetrical aryl(TMP)iodonium tosylate salts. The aryl transfer from the iodonium moiety occurs under electronic control with the electron-rich trimethoxyphenyl group acting as a competent dummy ligand. The yields of *N*-aryl phthalimides are moderate to high and the coupling reaction is compatible with electron-deficient and sterically encumbered aryl groups.

## Introduction

**Scheme 1:**
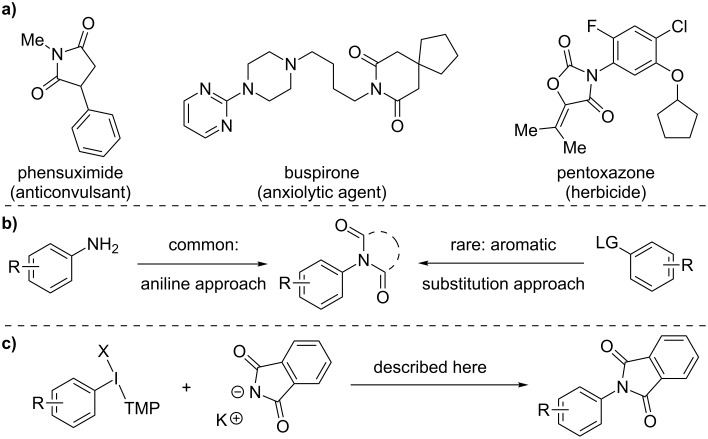
Imides as an important scaffold.

Imides are important structural units in a range of approved pharmaceuticals and agrochemicals (Scheme 1a) [1]. Despite the general prevalence of imides, *N*-aryl imide derivatives are relatively rare in such compounds. We were surprised by this disparity, but found that a lack of methods to synthesize *N*-aryl imides may explain their scarcity; this is particularly true relative to other *N*-aryl compounds. The survey of methods revealed that the dominant approach to *N*-aryl imides is to employ aniline starting materials (Scheme 1b, left), as was done in the synthesis of pentoxazone and related herbicides [2]. The alternative aromatic substitution approach with imide anions (Scheme 1b, right) is hampered by their low nucleophilicity [3]. Therefore, transition metals feature prominently in such methods, but even recent examples employ stoichiometric metal mediators [4]. Metal-free methods by classic S_N_Ar are also attractive, but only possible on very electron-deficient arene substrates [5]. Diaryliodonium salts are useful reagents for metal-free aryl transfer [6–10] and Muñiz and co-workers have recently reported an elegant study on sterically controlled C–N coupling of 2,6-disubstituted aryl(phenyl)iodonium salts and imides [11]. We have been investigating the generality of electronically controlled aryl transfer from aryl(trimethoxyphenyl)iodonium salts [12–14] and describe here the development of a C–N coupling of a phthalimide anion with non-sterically biased aryl groups. The protocol is compatible with *ortho*-, *meta*-, and *para-*substitution on the aryl group and the phthalimide moiety may also provide access to anilines.

## Results and Discussion

We initiated our optimization of the arylation of the potassium phthalimide nucleophile with diaryliodonium electrophiles by surveying several reaction conditions: dummy ligand (Aux), counter anion, solvent and volume, reaction temperature, and stoichiometry (Table 1). Consistent with an electronically controlled aryl transfer, the trimethoxyphenyl (TMP) auxiliary was superior to mesityl (Mes), phenyl (Ph), and anisyl (An) auxiliaries under several different reaction conditions (Table 1, entries 1–3; 11 and 12; 16 and 17). While the counter anion did not exert a dramatic influence on the reaction yield, using tosylate (OTs) produced the highest yield in both DCE and toluene as solvent (Table 1, entries 4–8). Given our ability to readily access aryl(TMP)iodonium tosylate salts [12] we continued our optimization with these reagents. We observed a very narrow operating temperature with a maximum yield at 100 °C when toluene was used as solvent (Table 1, entries 9, 10, and 12). We also observed that the reaction yield decreases with dilution (Table 1, entries 13–15). Finally, the yield increases with increasing stoichiometry of phthalimide (Table 1, entries 10, 13, and 17). It is also important to note that under “optimal” conditions (Table 1, entry 17) we did not observe a Phth–TMP adduct. Moreover, we did observe essentially quantitative formation of TMP–I and therefore complete consumption of **1a** and high fidelity for aryl transfer selectivity. At this time we are unable to account for the remaining mass balance (≈25%) of the methyl benzoate moiety of **1a**. We have employed the conditions of entry 17 (Table 1) as our standard conditions to evaluate the scope of this reaction.

**Table 1:** Discovery and optimization of reaction conditions.^a^

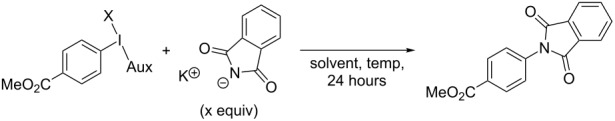						

Entry	X group	Aux group	Phth. equiv	Solvent	Temp. (°C)	^1^H NMR yield

1	TFA	Mes	2	DCE (1 mL)	70	27%
2	TFA	Ph	2	DCE (1 mL)	70	50%
3	TFA	TMP	2	DCE (1 mL)	70	52%
4	TFA	TMP	3	DCE (1 mL)	80	54%
5	OTs	TMP	3	DCE (1 mL)	80	62%
6	TFA	TMP	3	toluene (0.42 mL)	100	50%
7	OTf	TMP	3	toluene (0.42 mL)	100	56%
8	OTs	TMP	3	toluene (0.42 mL)	100	68%
9	OTs	TMP	3	toluene (0.5 mL)	90	64%
10	OTs	TMP	3	toluene (0.5 mL)	100	70%
11	OTs	Ph	3	toluene (0.5 mL)	100	23%
12	OTs	TMP	3	toluene (0.5 mL)	110	62%
13	OTs	TMP	1.1	toluene (0.5 mL)	100	39%
14	OTs	TMP	1.1	toluene (1 mL)	100	28%
15	OTs	TMP	1.1	toluene (1.5 mL)	100	16%
16	OTs	An	5	toluene (0.5 mL)	100	46%
17	OTs	TMP	5	toluene (0.5 mL)	100	75%

^a^Conditions: **1** (0.1 mmol, 1 equiv), potassium phthalimide (see table for equivalents), solvent (see table), temperature (see table), 24 hours.

**Scheme 2:**
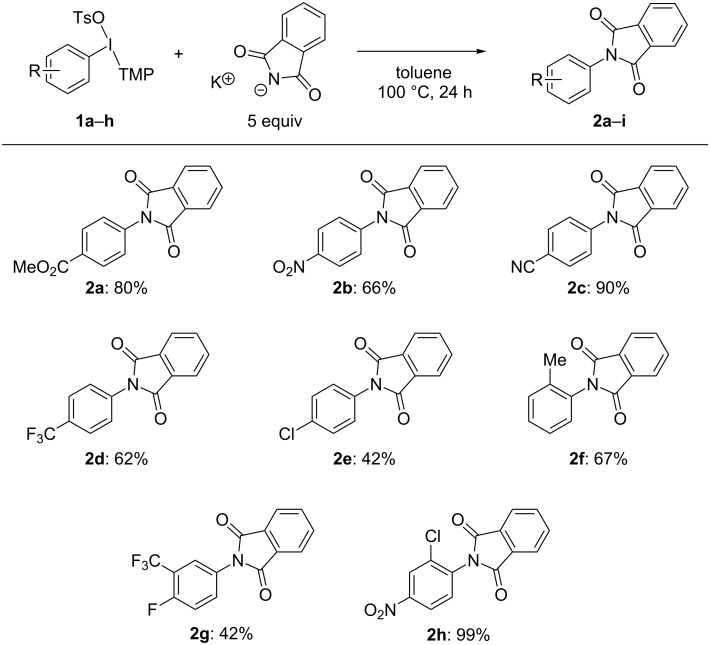
Scope of compatible aryl groups. Conditions: **1** (0.5 mmol, 1 equiv), potassium phthalimide (2.5 mmol, 5 equiv), toluene (2.5 mL), 100 °C, 24 hours. Isolated yields are reported.

We have assessed the scope of compatible aryl groups under our optimal conditions (Scheme 2). Under electronic control, strong electron-withdrawing groups on the aryl ring lead to high yield of *N*-aryl phthalimide products (Scheme 2, **2a**–**d**, 66–90% yield). However, the TMP auxiliary also enables the coupling of phthalimide with moderately electron-deficient aryl groups. For instance the *p*-chlorophenyl moiety (**2e**) is coupled to phthalimide in moderate yield (42%). Additionally, we have observed that electronic and steric effects operate in concert to couple an *o*-tolyl (**2f**) moiety to phthalimide in high yield (67%). In this case, 2,6-disubstituted aryl groups are not required for a sterically controlled coupling. Finally, as an example two polysubstituted aryl groups are introduced in this coupling reaction, which are specifically enabled by the use of an unsymmetrical aryl(TMP)iodonium electrophile (Scheme 2, **2g** and **2h**, 42 and 99% yield, respectively).

**Scheme 3:**
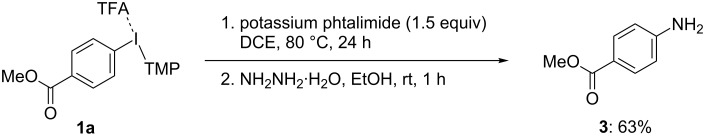
One-pot synthesis of anilines.

The phthalimide moiety is well-recognized as an “NH_3_” surrogate, and the products depicted in Scheme 2 may be deprotected to yield aniline derivatives. In a specific example, **1a** is reacted under modified conditions to yield **2a**. In this one-pot procedure, hydrazine in aqueous ethanol is added directly to the reaction mixture and aniline **3** is isolated in 63% yield from **1a** (Scheme 3).

We have previously described the coupling of aryl(TMP)iodonium tosylates with azide nucleophiles [14]. Azide is a notably stronger nucleophile than phthalimide and it is interesting to compare the reaction of these two nucleophiles with **1a** under similar conditions (Table 2). The Mayr nucleophilicity constant of azide [15] is 20.5 and high yield (95%) is observed in a reaction with **1a** under relatively mild temperature (65 °C) and short reaction time (2 hours, Table 2, entry 1). The Mayr nucleophilicity constant for phthalimide is five-orders of magnitude lower (15.5) [3] and under similar conditions leads to trace product (Table 2, entry 2). In order to obtain a high yield of **2a**, albeit lower than given in entry 1, a higher temperature (100 °C) and a longer reaction time (24 hours) are required (Table 2, entry 3). This suggests that the contribution of nucleophilicity (via Mayr nucleophilicity constants) [16] may be useful in developing other coupling reactions with diaryliodonium electrophiles.

**Table 2:** Comparison of nucleophilicity.

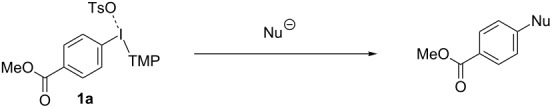					

Entry	Nucleophile (Nu)	Mayr nucleophilicity	Temperature (°C)	Time	Yield

1	azide	20.5	65	2 hours	95%
2	phthalimide	15.5	65	2 hours	trace
3	phthalimide	15.5	100	24 hours	80%

## Conclusion

The coupling of both electron-deficient and sterically encumbered aryl groups with a phthalimide anion is achievable with aryl(TMP)iodonium tosylate salts. This is an electronically controlled coupling reaction that is enabled by the TMP auxiliary and complementary to the sterically controlled coupling previously reported. We anticipate that this reaction will find use as a starting point for the synthesis of *N*-aryl imides in a range of applications.

## Supporting Information

File 1 General experimental details, procedures, tabulated spectroscopic data, and ^1^H, ^13^C{^1^H}, and ^19^F NMR spectra of compounds **1g**, **2a**–**i**, and **3**.
