# Supplementary material for: Imide arylation with aryl(TMP)iodonium tosylates
Source: Beilstein J Org Chem. 2018 May 11;14:1034–8. doi: 10.3762/bjoc.14.90 (PMC6009222; doi:10.3762/bjoc.14.90)
Supplement: File 1 — General experimental details, procedures, tabulated spectroscopic data, and 1H, 13C{1H}, and 19F NMR spectra of compounds 1g, 2a–i, and 3. [file Beilstein_J_Org_Chem-14-1034-s001.pdf]

Supporting Information

for

Imide arylation with aryl(TMP)iodonium tosylates

Souradeep Basu, Alexander H. Sandtorv and David R. Stuart*

Address: Department of Chemistry, Portland State University, Portland OR 97201, United States

Email: David Stuart - dstuart@pdx.edu

* Corresponding author

General experimental details, procedures, tabulated spectroscopic data, and ^{1}H, ^{13}C{^{1}H}, and ^{19}F NMR spectra of compounds 1g, 2a–i, and 3

Table of Contents

General consideration… S2

Synthesis and characterization of products… S2–S6

^{1}H and ^{13}C spectra… S7–S17

References… S18

General considerations.

Commercially available reagents and solvents were used without further purification unless otherwise stated. *m*-CPBA was assayed by iodometric titration and found to contain 72% active oxidant. Crude reaction mixtures were analyzed by ^{1}H NMR spectroscopy and thin-layer chromatography (TLC) on silica gel (60 Å F-254) TLC plates and visualized by UV irradiation, and in some cases using a $KMnO_4$ stain. Crude materials were purified by flash column chromatography on silica gel unless otherwise stated. ^{1}H, ^{13}C{^{1}H}, ^{19}F{^{1}H} NMR spectra were recorded in $CDCl_3$ or DMSO-d_6 (referenced to the solvent peak) on a 400 MHz spectrometer at 298 K unless otherwise stated. The following notation is used: s, singlet; d, doublet; t, triplet; q, quartet; m, multiplet; dd, doublet of doublets. FTIR spectra were obtained from solutions in DCM on NaCl plates and the following notation is used: w, weak; m, medium; s, strong; br, broad. High-resolution mass spectrometry (HRMS) data were obtained by electrospray ionization (ESI) with an ion trap mass analyzer or electron impact (EI, 70 eV). Melting points are reported as uncorrected.

Compounds **1a**–**e**, **g**, and **h** were prepared according to previously reported methods.[1]

Synthesis and characterization of products

A: General procedure for synthesis of aryl(TMP)iodonium tosylates:

Aryl iodide (5 mmol, 1 equiv) and acetonitrile (5 mL) were added to a round bottom flask, equipped with a magnetic stir bar. Toluenesulfonic acid (5.05 mmol, 1.01 equiv) was added in one portion followed by one portion of *m*-CPBA (5.05 mmol, 1.01 equiv). The reaction mixture was stirred at room temperature until a white solid was formed. After 1 h 1,3,5-trimethoxybenzene (5.05 mmol, 1.01 equiv) was added and stirred until it forms clear solution. The reaction mixture was triturated with diethyl ether. The precipitate was isolated by vacuum filtration and washed with diethyl ether (3 × 20 mL). After drying under high vacuum the diaryliodonium salt was obtained in analytical pure form.

B: General Procedure for the reaction of aryl(TMP)iodonium tosylate with potassium phthalimide:

Aryl(trimethoxyphenyl)iodonium tosylate salt (0.5 mmol, 1 equiv) was transferred to a 5 mL vial equipped with magnetic stirring bar, followed by the addition of potassium phthalimide (2.5 mmol, 5 equiv). The vial was sealed with a screw cap and toluene (2.5 mL) was transferred to the vial by means of syringe through septa. The vial was heated to 100 °C for 24 h in an aluminum block. The post reaction mixture was cooled and quenched with saturated aqueous KOH solution. Then the reaction mixture was extracted with DCM (3 times) and the total organic portion was dried with anhydrous $MgSO_4$. The solvent was evaporated under reduced pressure and the crude was purified through flash column chromatography using a gradient eluent composed of diethyl ether in hexanes (10–40%).

Compound 1f: *o*-tolyl(2,4,6-trimethoxyphenyl)iodonium tosylate

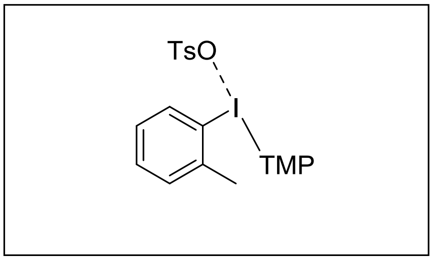

Prepared according to the general procedure A on 5 mmol and obtained an isolated yield of 96% (2.670 g) as white solid.

^{1}H NMR (400 MHz, DMSO-d_6) δ 8.06 (d, *J* = 8.0 Hz, 1H), 7.52 (t, *J* = 2.6 Hz, 2H), 7.47 (dd, *J* = 8.0, 1.8 Hz, 2H), 7.24 (tt, *J* = 6.2, 2.7 Hz, 1H), 7.11 (d, *J* = 7.7 Hz, 2H), 6.45 (s, 2H), 3.98 (s, 6H), 3.86 (s, 3H), 2.61 (s, 3H), 2.29 (s, 3H).

^{13}C NMR (101 MHz, DMSO) δ 166.4, 159.8, 146.2, 140.9, 138.0, 137.5, 132.7, 131.5, 129.3, 128.4, 125.9, 121.8, 92.5, 87.1, 57.6, 56.6, 25.2, 21.2.

FTIR: 2986, 2364, 2340, 1582, 1453, 1430, 1410, 1342, 1219, 1178, 1160, 1033, 1006 cm^{-1}

HRMS: Calculated for $C_{16}H_{18}IO_3^+$ $[M\text{-}OTs]^+$: 385.0295; Observed: 385.0336

Melting point: 173-174 °C

Compound 2a: methyl 4-(1,3-dioxoisoindolin-2-yl)benzoate

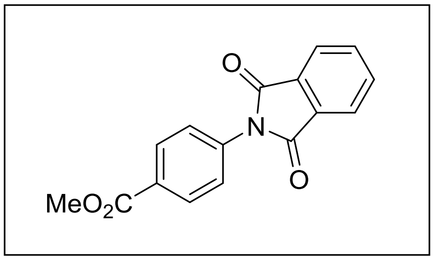

Prepared according to the general procedure B on 0.5 mmol and obtained an isolated yield of 80% (0.112 g) as white solid. Spectral data is consistent with that previously reported.[2]

^1H NMR (400 MHz, $CDCl_3$) δ 8.18 (d, *J* = 8.7 Hz, 2H), 7.97 (dd, *J* = 5.5, 3.1 Hz, 2H), 7.82 (dd, *J* = 5.5, 3.1 Hz, 2H), 7.6 (d, *J* = 8.7 Hz, 2H), 3.94 (s, 3H).

^{13}C NMR (101 MHz, $CDCl_3$) δ 166.9, 166.4, 136.0, 134.8, 131.7, 130.5, 129.4, 126.0, 124.0, 52.4.

Compound 2b: 2-(4-nitrophenyl)isoindoline-1,3-dione

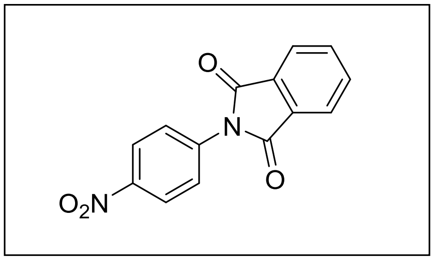

Prepared according to the general procedure B on 0.5 mmol and obtained an isolated yield of 66% (0.089 g) as white solid. Spectral data is consistent with that previously reported.[3]

^1H NMR (400 MHz, $CDCl_3$) δ 8.38 (d, *J* = 9.1 Hz, 2H), 8.00 (dd, *J* = 5.5, 3.1 Hz, 2H), 7.85 (dd, *J* = 5.5, 3.1 Hz, 2H), 7.78 (d, *J* = 9.1 Hz, 2H).

^{13}C NMR (101 MHz, $CDCl_3$) δ 166.4, 146.4, 137.5, 135.0, 131.3, 126.3, 124.4, 124.2.

Compound 2c: 4-(1,3-dioxoisoindolin-2-yl)benzonitrile

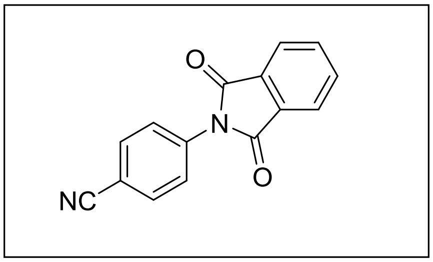

Prepared according to the general procedure B on 0.5 mmol and obtained an isolated yield of 90% (0.111 g) as white solid. Spectral data is consistent with that previously reported.[4]

^{1}H NMR (400 MHz, $CDCl_3$) δ 7.99 (dd, J = 5.5, 3.1 Hz, 2H), 7.84 (dd, J = 5.5, 3.1 Hz, 2H), 7.80 (d, J = 8.7 Hz, 2H), 7.69 (d, J = 8.7 Hz, 2H).

^{13}C NMR (101 MHz, $CDCl_3$) δ 166.4, 135.9, 134.9, 132.9, 131.4, 126.5, 124.1, 118.3, 111.3.

Compound 2d: 2-(4-(trifluoromethyl)phenyl)isoindoline-1,3-dione

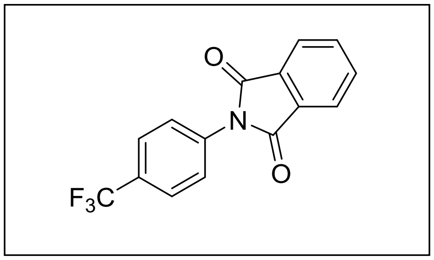

Prepared according to the general procedure B on 0.5 mmol and obtained an isolated yield of 62% (0.090 g) as yellowish white solid. Spectral data is consistent with that previously reported.[5]

^{1}H NMR (400 MHz, $CDCl_3$) δ 7.99 (dt, J = 5.0, 2.0 Hz, 2H), 7.83 (dt, J = 5.1, 1.9 Hz, 2H), 7.78 (d, J = 8.3 Hz, 2H), 7.65 (d, J = 8.2 Hz, 2H).

^{13}C NMR (101 MHz, $CDCl_3$) δ 166.7, 135.7, 134.7, 131.5, 129.8 (q, J = 128 Hzz), 126.4, 126.2 (q, J = 3.8 Hz), 125.1, 124.0 (d, J = 2.7 Hz).

^{19}F NMR (376 MHz, Chloroform-*d*) δ -62.62.

Compound 2e: 2-(4-chlorophenyl)isoindoline-1,3-dione

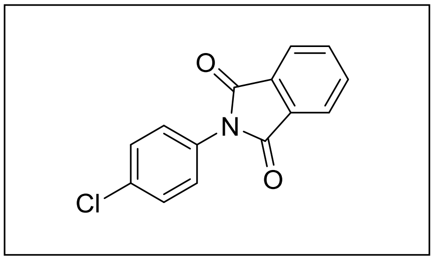

Prepared according to the general procedure B on 0.5 mmol and obtained an isolated yield of 42% (0.054 g) as white solid. Spectral data is consistent with that previously reported.[6]

^{1}H NMR (400 MHz, $CDCl_3$) δ 7.96 (dd, *J* = 5.5, 3.0 Hz, 2H), 7.81 (dd, *J* = 5.5, 3.0 Hz, 2H), 7.48 (d, *J* = 8.8 Hz, 2H), 7.42 (d, *J* = 8.8 Hz, 2H).

^{13}C NMR (101 MHz, $CDCl_3$) δ 166.9, 134.5, 133.8, 131.6, 130.2, 129.3, 127.6, 123.8.

Compound 2f: 2-(*o*-tolyl)isoindoline-1,3-dione

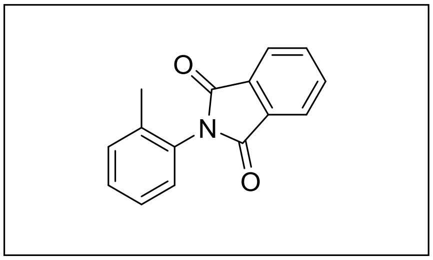

Prepared according to the general procedure B on 0.5 mmol and obtained an isolated yield of 67% (0.080 g) as yellowish white solid. Spectral data is consistent with that previously reported.[3]

^{1}H NMR (400 MHz, $CDCl_3$) δ 8.11 – 7.87 (m, 2H), 7.87 – 7.68 (m, 2H), 7.43 – 7.29 (m, 3H), 7.20 (d, *J* = 7.6 Hz, 1H), 2.21 (s, 3H).

^{13}C NMR (101 MHz, $CDCl_3$) δ 167.3, 136.5, 134.3, 132.0, 131.2, 130.6, 129.5, 128.7, 126.8, 123.7, 18.0.

Compound 2g: 2-(4-fluoro-3-(trifluoromethyl)phenyl)isoindoline-1,3-dione

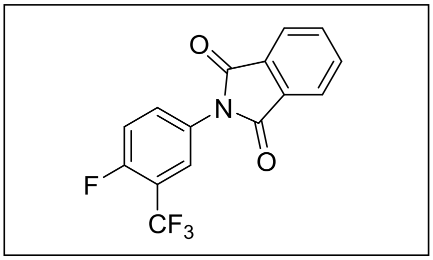

Prepared according to the general procedure B on 0.5 mmol and obtained an isolated yield of 42% (0.065 g) as yellowish white solid.

^{1}H NMR (400 MHz, $CDCl_3$) δ 8.02 – 7.94 (m, 2H), 7.83 (dt, *J* = 5.3, 2.4 Hz, 2H), 7.76 (dd, *J* = 6.0, 2.6 Hz, 1H), 7.68 (dp, *J* = 6.5, 2.2 Hz, 1H), 7.35 (t, *J* = 9.3 Hz, 1H).

^{13}C NMR (101 MHz, $CDCl_3$) δ 166.7, 159.9, 157.4, 134.8, 131.9 (d, *J* = 9.0 Hz), 131.4, 127.8 (d, *J* = 3.7 Hz), 126.6 – 124.8 (m), 124.0, 119.2 (dd, *J* = 33.6, 13.8 Hz), 117.8 (d, *J* = 84 Hz).

^{19}F NMR (376 MHz, Chloroform-*d*) δ -61.59 (d, *J* = 12.7 Hz), -114.45 – -114.52 (m).

FTIR: 2917, 2845, 1718, 1503, 1434, 1321, 1242, 1110 cm^{-1}

GCMS: Calculated for $C_{15}H_7F_4NO_2$, 309.22; Observed, 309.10

Melting Point: 163.9 ^{o}C

Compound 2h: 2-(2-chloro-4-nitrophenyl)isoindoline-1,3-dione

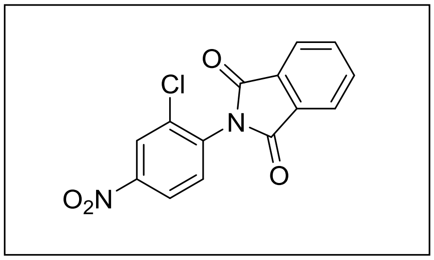

Prepared according to the general procedure B on 0.5 mmol and obtained an isolated yield of 99% (0.150 g) as white solid. Spectral data is consistent with that previously reported.[7]

^{1}H NMR (400 MHz, $CDCl_3$) δ 8.46 (d, J = 2.5 Hz, 1H), 8.30 – 8.25 (m, 1H), 8.03 – 7.96 (m, 2H), 7.89 – 7.82 (m, 2H), 7.57 (d, J = 8.7 Hz, 1H).

^{13}C NMR (101 MHz, $CDCl_3$) δ 165.8, 148.5, 135.8, 135.1, 134.8, 131.8, 131.6, 125.9, 124.5, 122.7.

Compound 3: methyl 4-aminobenzoate

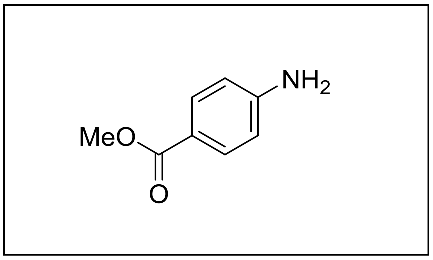

To a 5-mL vial equipped with a magnetic stirring bar was transferred diaryliodonium trifluoroacetate salt (0.2 mmol), followed by potassium phthalimide (0.056 g, 0.30 mmol). The vial was sealed with a screw cap and dichloroethane (1 mL) was transferred to the vial by means of syringe through the septa. The vial was heated to 80 ^oC for 24 h in an aluminum block.

The reaction mixture was cooled to room temperature. Ethanol (1 mL) was added, followed by $NH_2NH_2 \cdot H_2O$ (28 µL) both by means of syringes through the septa. The mixture was stirred at room temperature. After 30 minutes, a new portion of by $NH_2NH_2 \cdot H_2O$ (28 µL) was added and the mixture was allowed to stir for an additional 60 minutes, leading to formation of a precipitate. The crude reaction mixture was filtered through a pad of silica, and the pad was washed with diethyl ether (3 × 1 mL). Silica-gel was added to the filtrate, and the solvent was removed under reduced pressure. The crude was purified by means of column chromatography using a gradient eluent composed of hexanes in diethyl ether (20% → 50% → 100%). The product was obtained as a white solid in a yield of 63% (0,019 g, 0.13 mmol). Spectral data is consistent with that previously reported.[8]

^{1}H NMR (400 MHz, $CDCl_3$) δ 7.85 (d, J = 8.7 Hz, 2H), 6.64 (d, J = 8.7 Hz, 2H), 4.05 (s, 2H), 3.85 (s, 3H).

^{13}C NMR (101 MHz, $CDCl_3$) δ 167.3, 150.9, 131.7, 119.9, 113.9, 51.8.

^{1}H, ^{13}C and ^{19}F NMR spectra

^{1}H NMR of o-tolyl(2,4,6-trimethoxyphenyl)iodonium tosylate at 400 MHz in $CDCl_3$ at 298 K

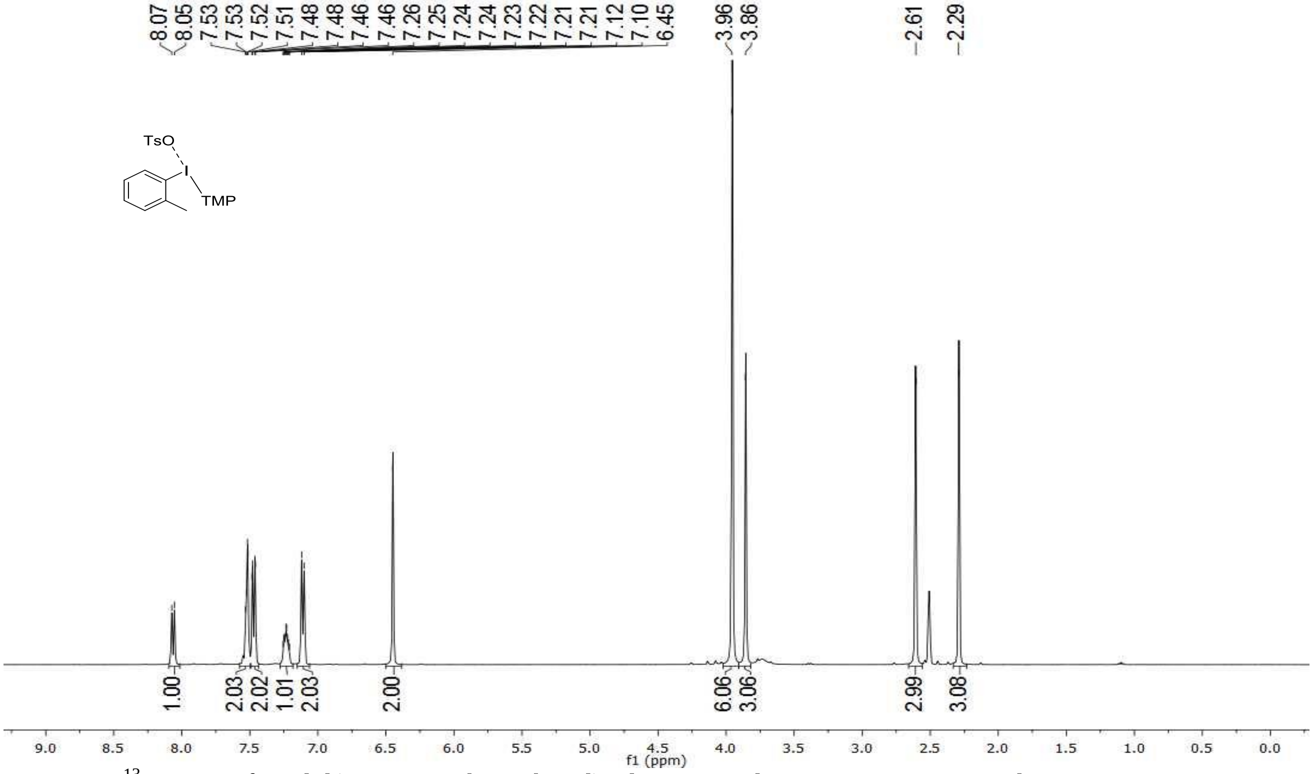

^{13}C NMR of o-tolyl(2,4,6-trimethoxyphenyl)iodonium tosylate at 101 MHz in $CDCl_3$ at 298 K

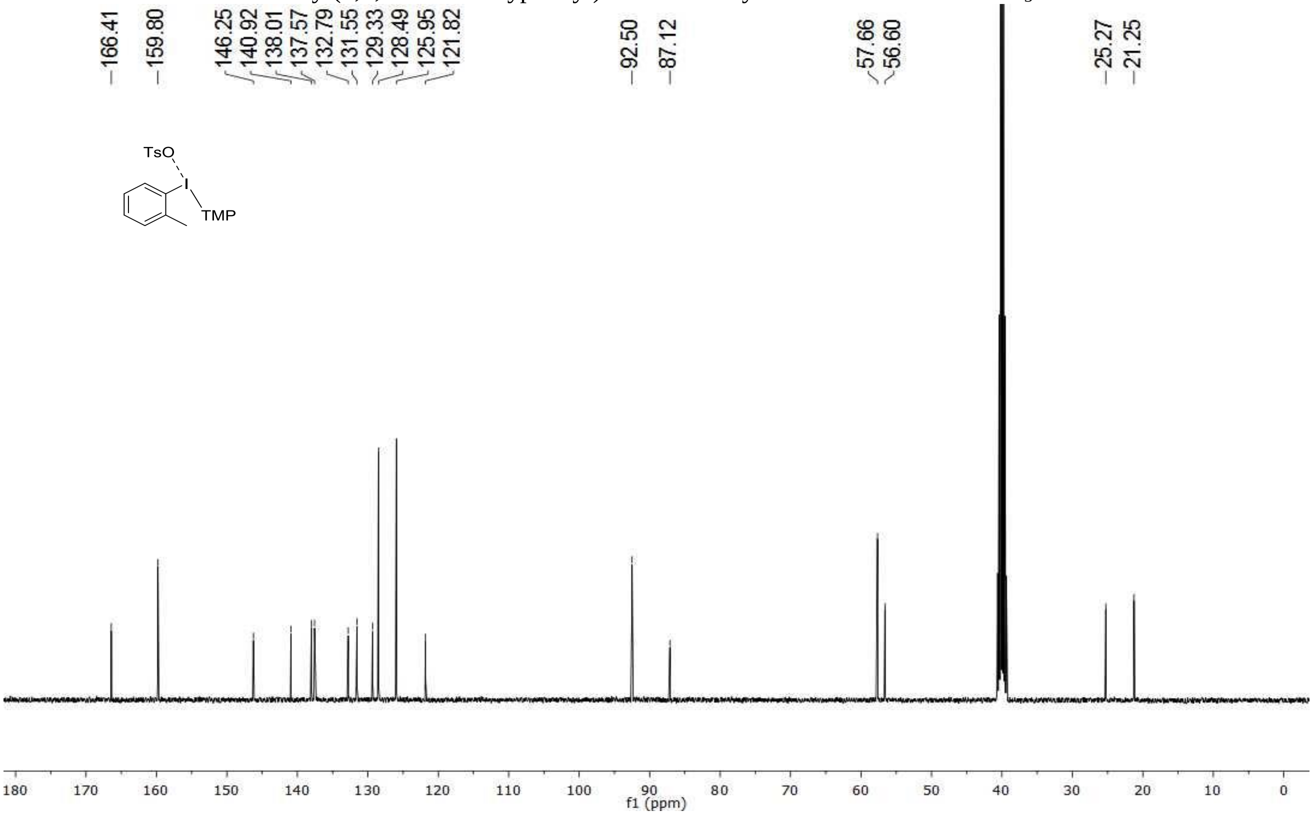

^{1}H NMR of methyl 4-(1,3-dioxoisoindolin-2-yl)benzoate at 400 MHz in $CDCl_3$ at 298 K

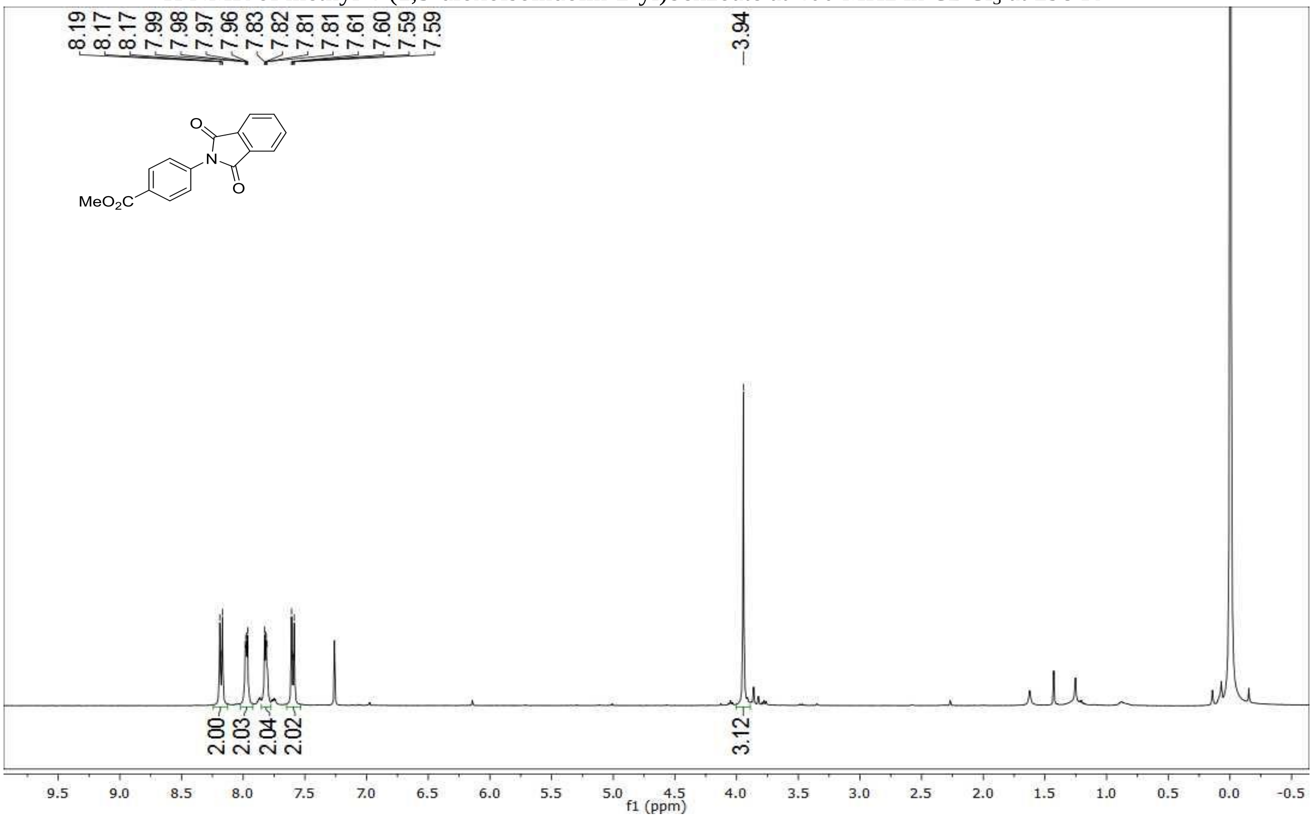

^{13}C NMR of methyl 4-(1,3-dioxoisoindolin-2-yl)benzoate at 101 MHz in $CDCl_3$ at 298 K

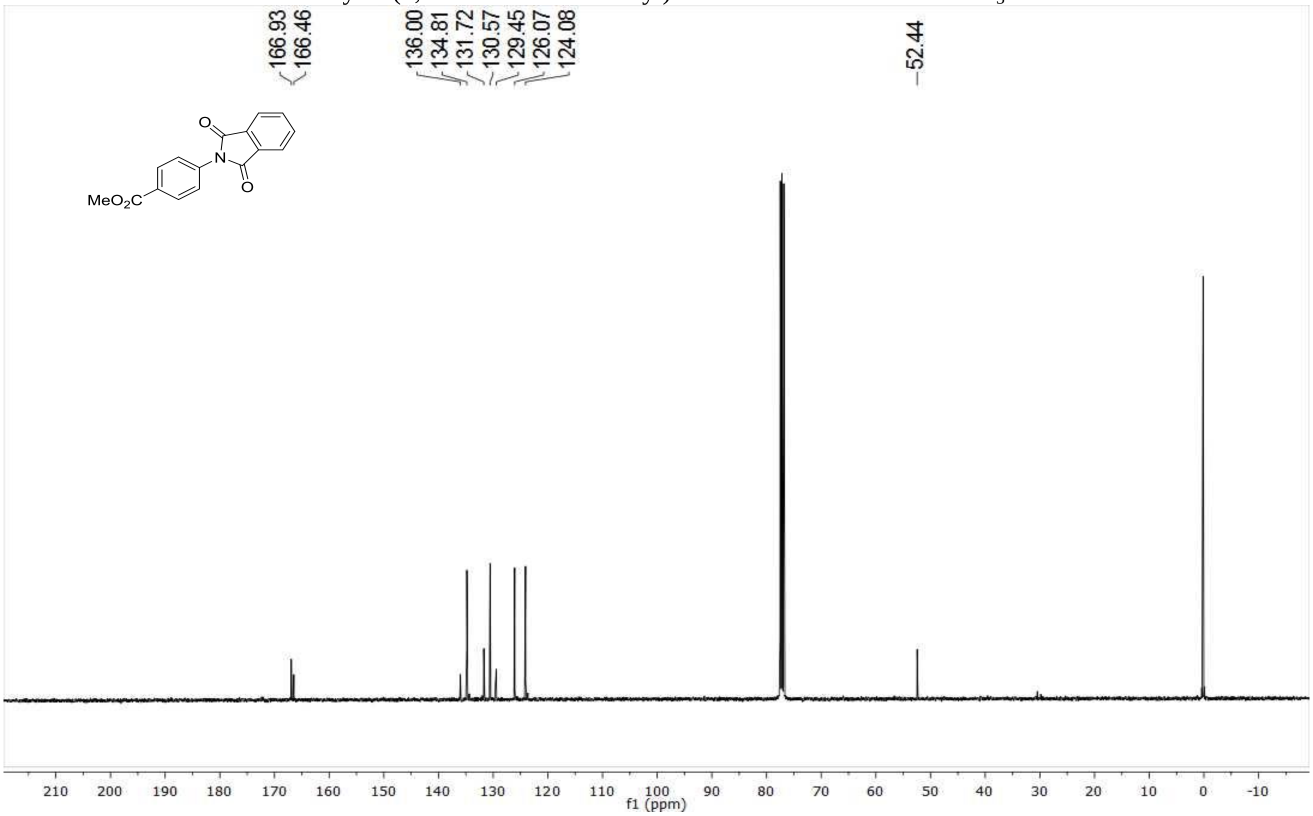

^{1}H NMR of 2-(4-nitrophenyl)isoindoline-1,3-dione at 400 MHz in $CDCl_3$ at 298 K

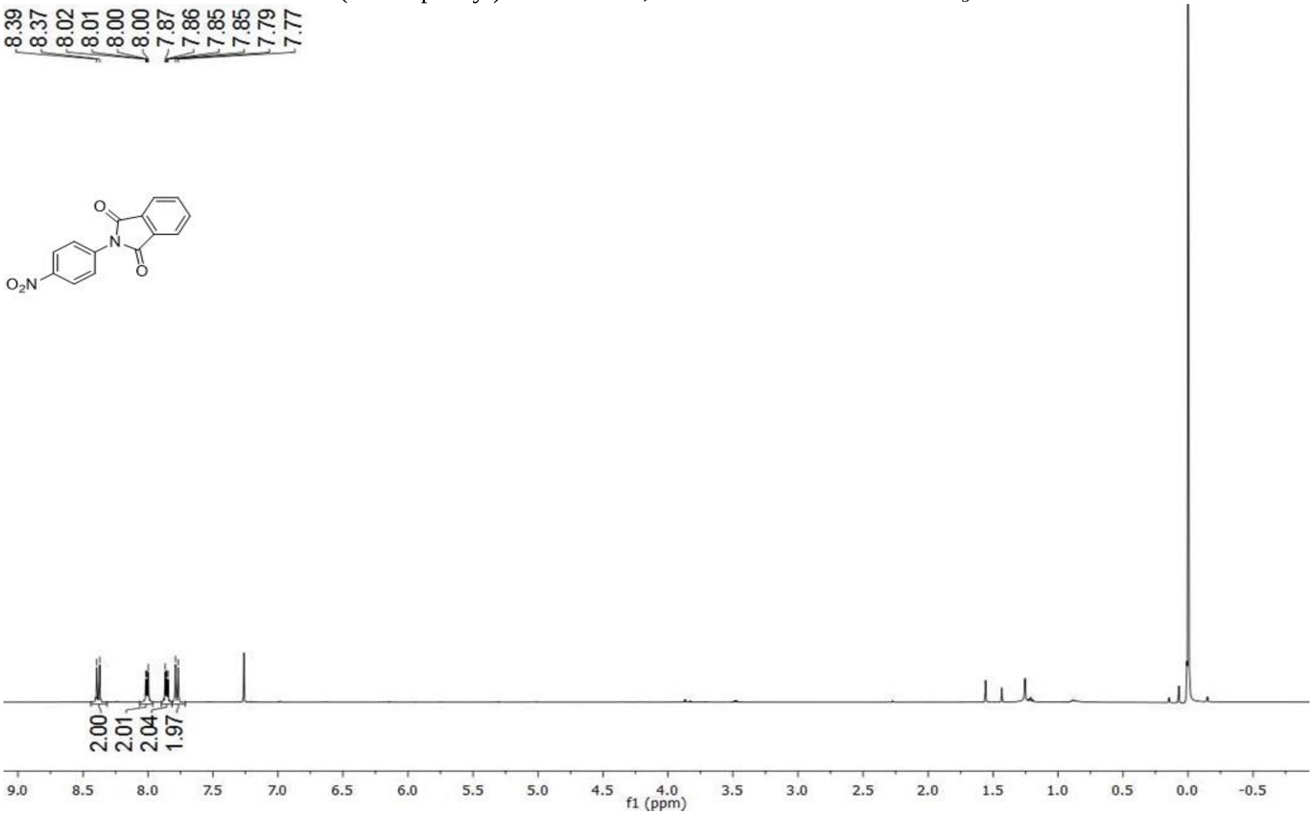

^{13}C NMR of 2-(4-nitrophenyl)isoindoline-1,3-dione at 101 MHz in $CDCl_3$ at 298 K

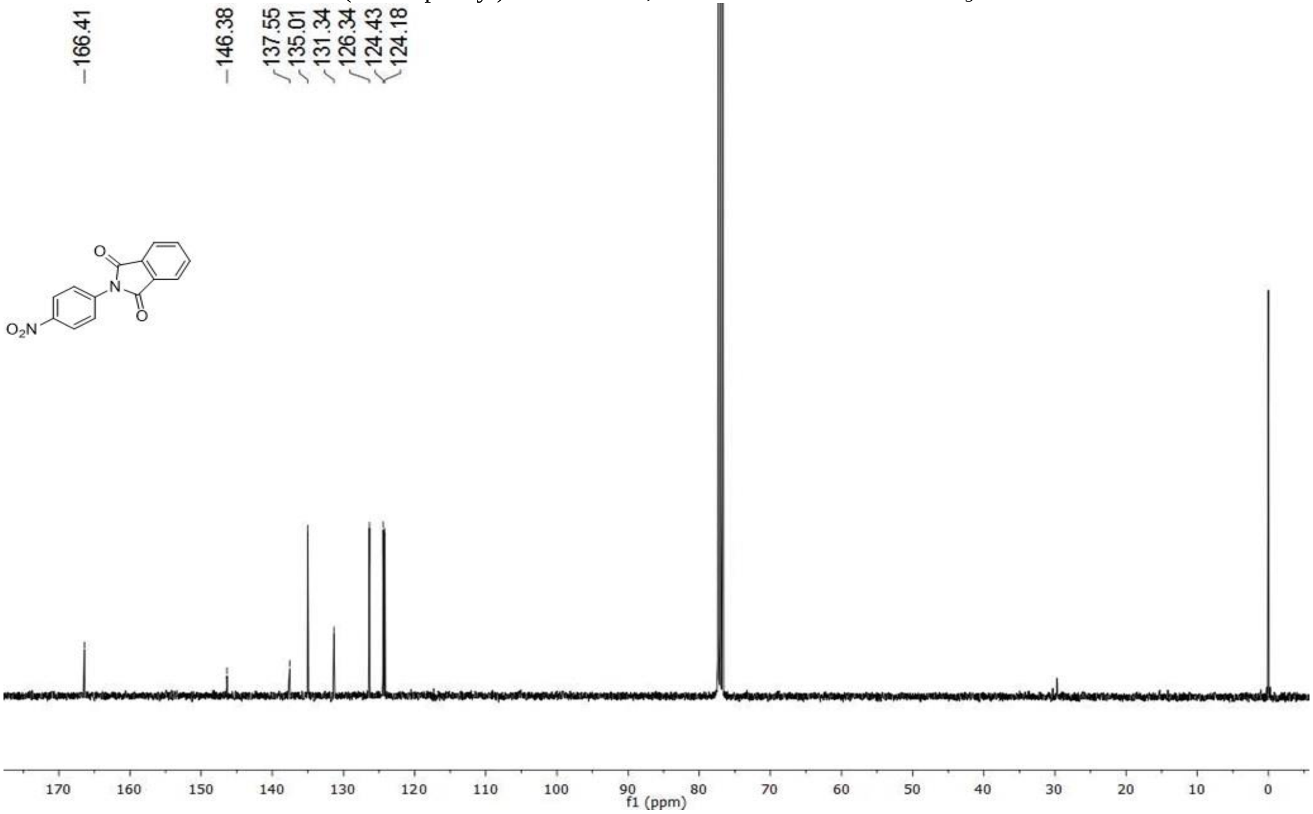

^{1}H NMR of 4-(1,3-dioxoisoindolin-2-yl)benzonitrile at 400 MHz in $CDCl_3$ at 298 K

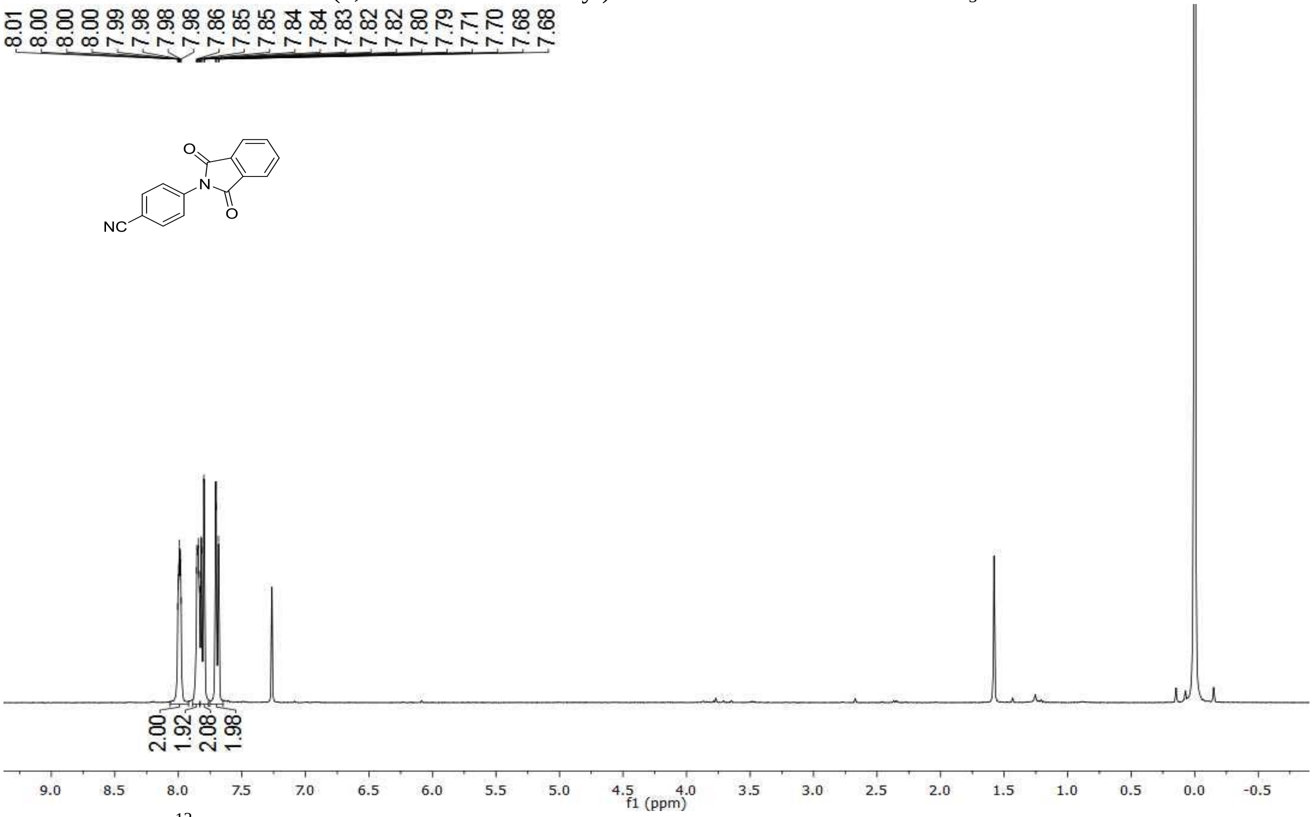

^{13}C NMR of 4-(1,3-dioxoisoindolin-2-yl)benzonitrile at 101 MHz in $CDCl_3$ at 298 K

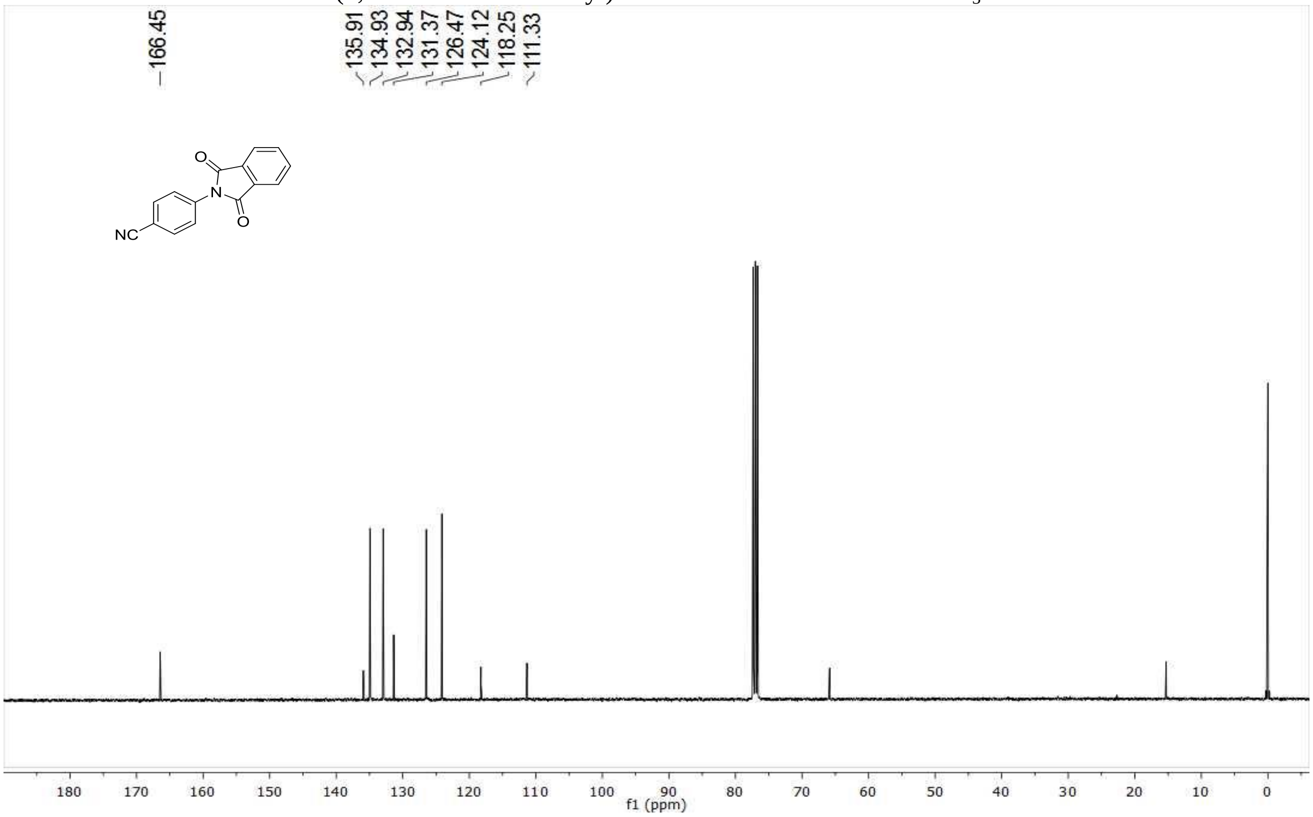

^{1}H NMR of 2-(4-(trifluoromethyl)phenyl)isoindoline-1,3-dione at 400 MHz in $CDCl_3$ at 298 K

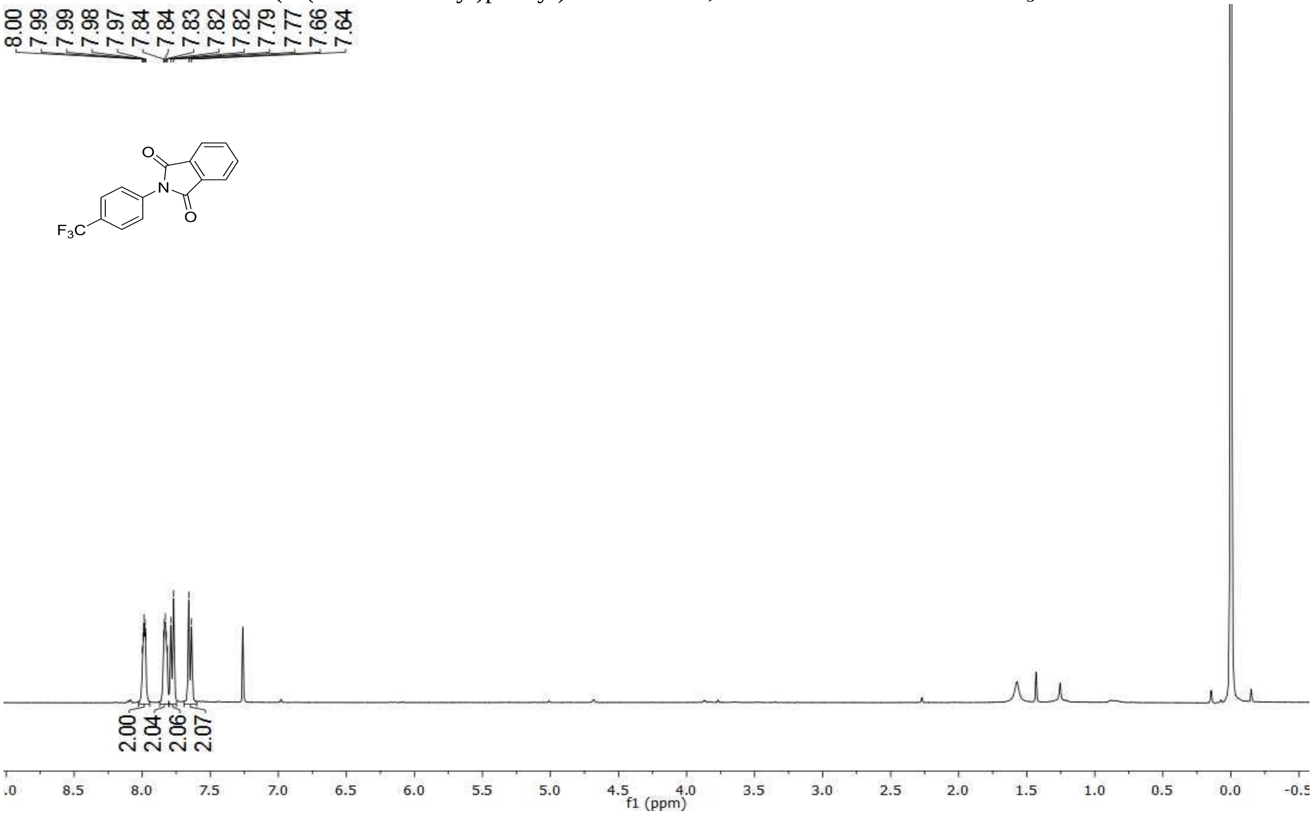

^{13}C NMR of 2-(4-(trifluoromethyl)phenyl)isoindoline-1,3-dione at 101 MHz in $CDCl_3$ at 298 K

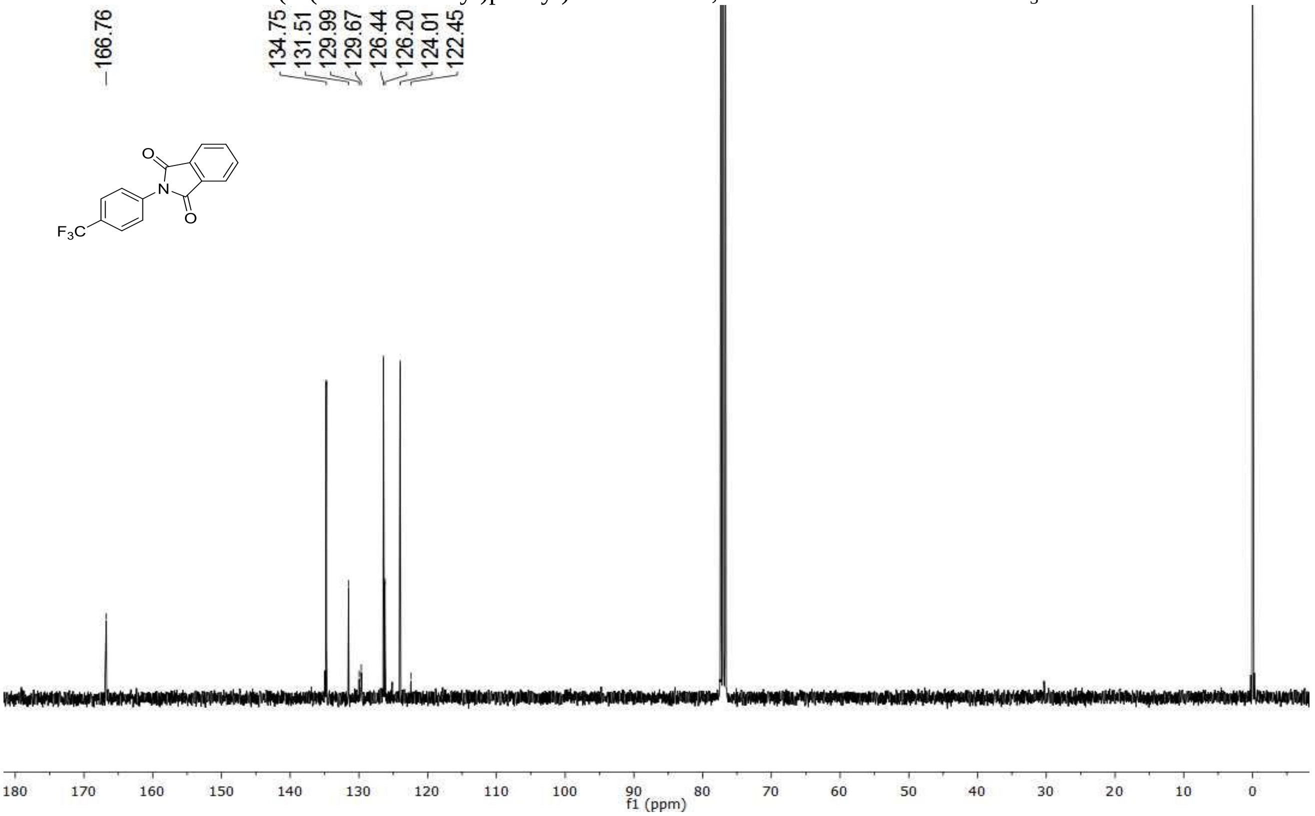

^{19}F NMR of 2-(4-(trifluoromethyl)phenyl)isoindoline-1,3-dione at 376 MHz in $CDCl_3$ at 298 K

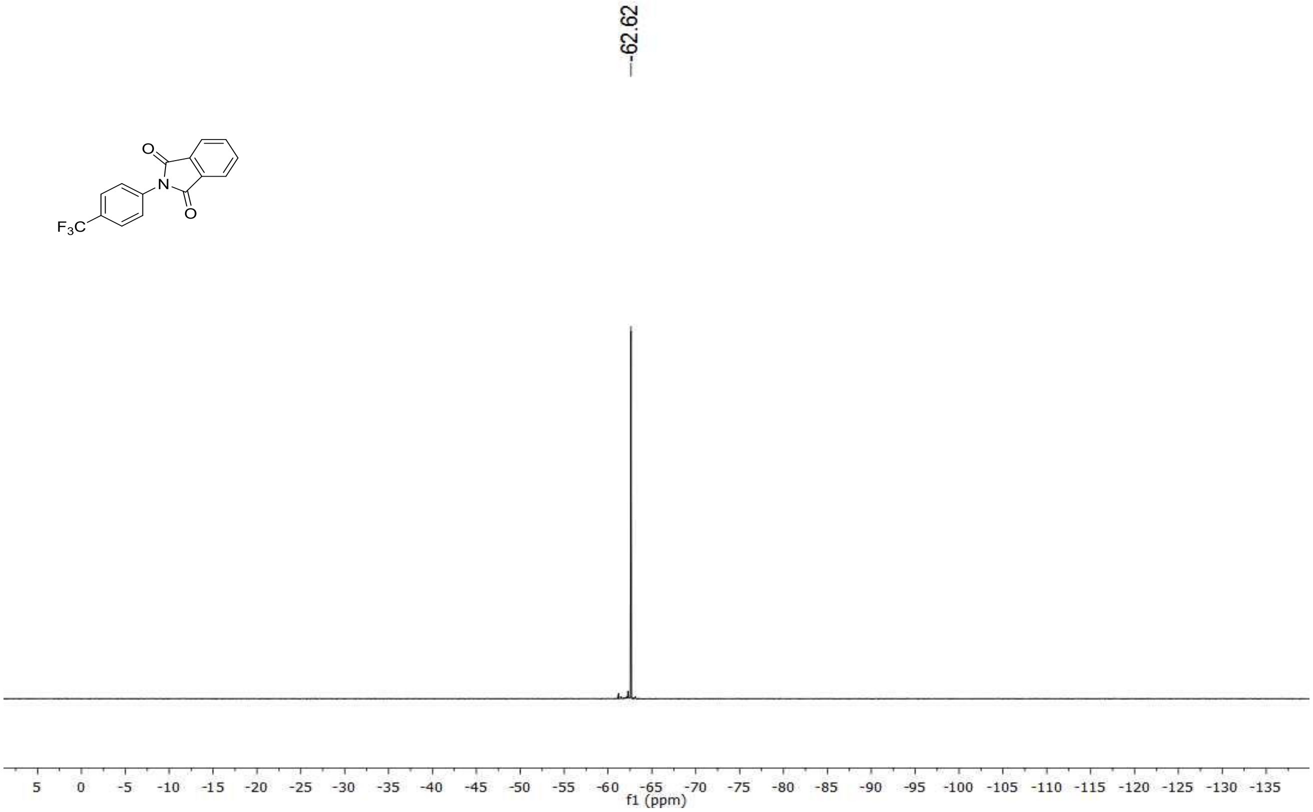

^{1}H NMR of 2-(4-chlorophenyl)isoindoline-1,3-dione at 400 MHz in $CDCl_3$ at 298 K

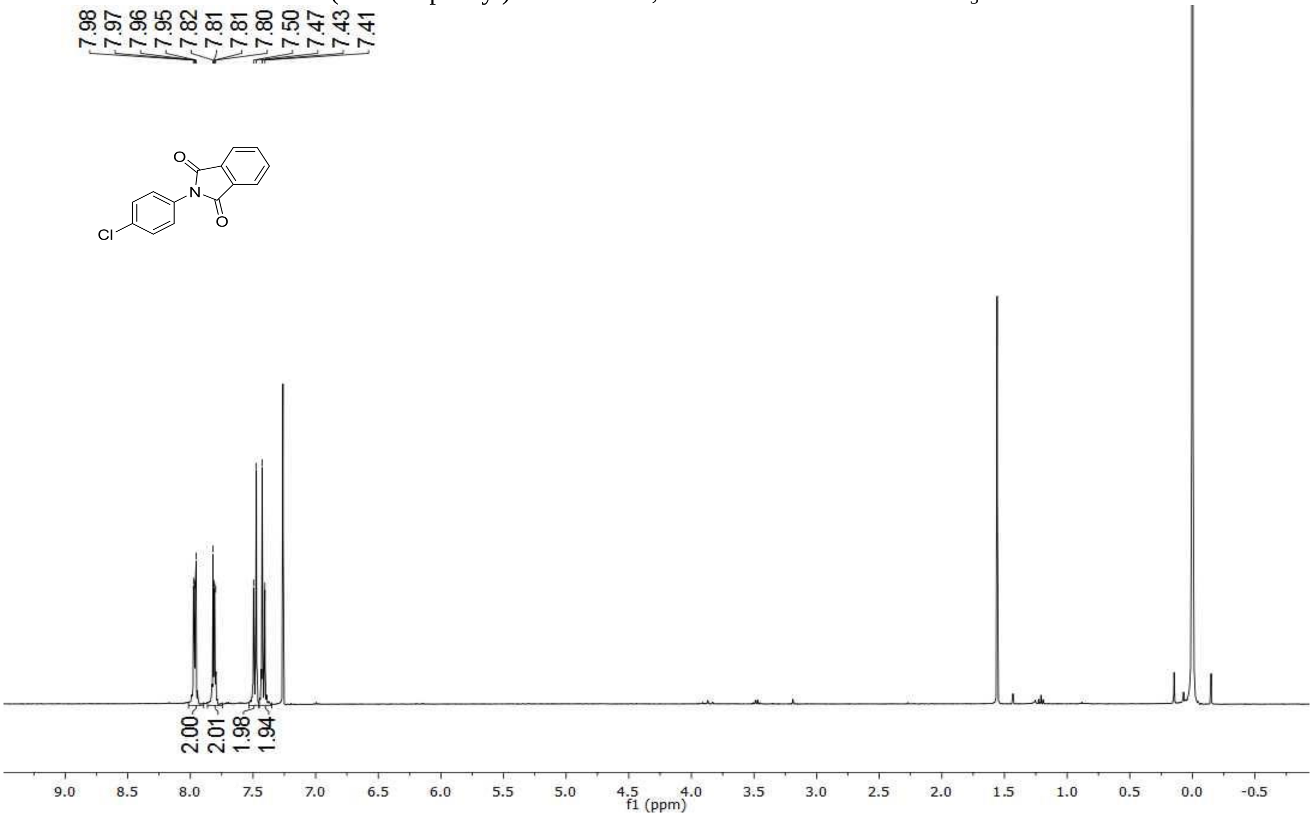

^{13}C NMR of 2-(4-chlorophenyl)isoindoline-1,3-dione at 101 MHz in $CDCl_3$ at 298 K

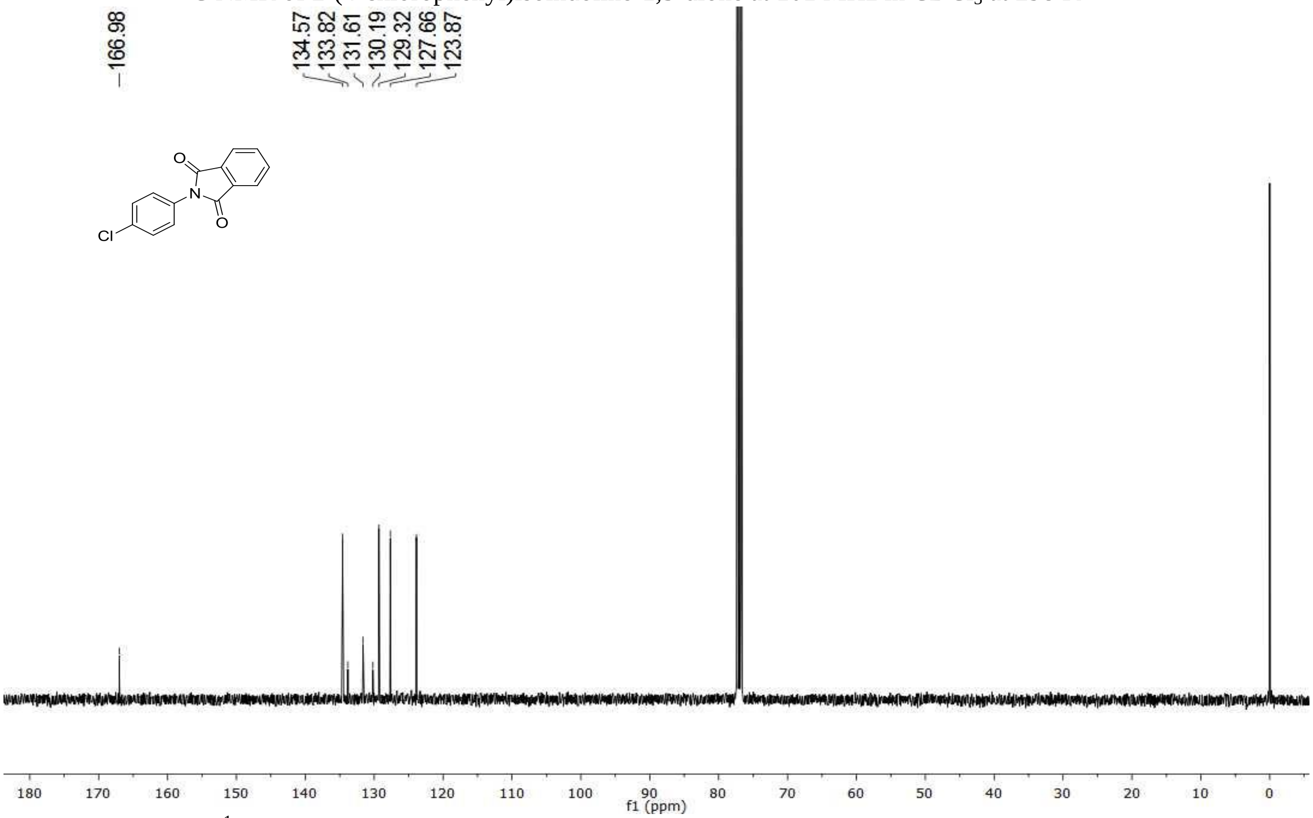

^{1}H NMR of 2-(o-tolyl)isoindoline-1,3-dione at 400 MHz in $CDCl_3$ at 298 K

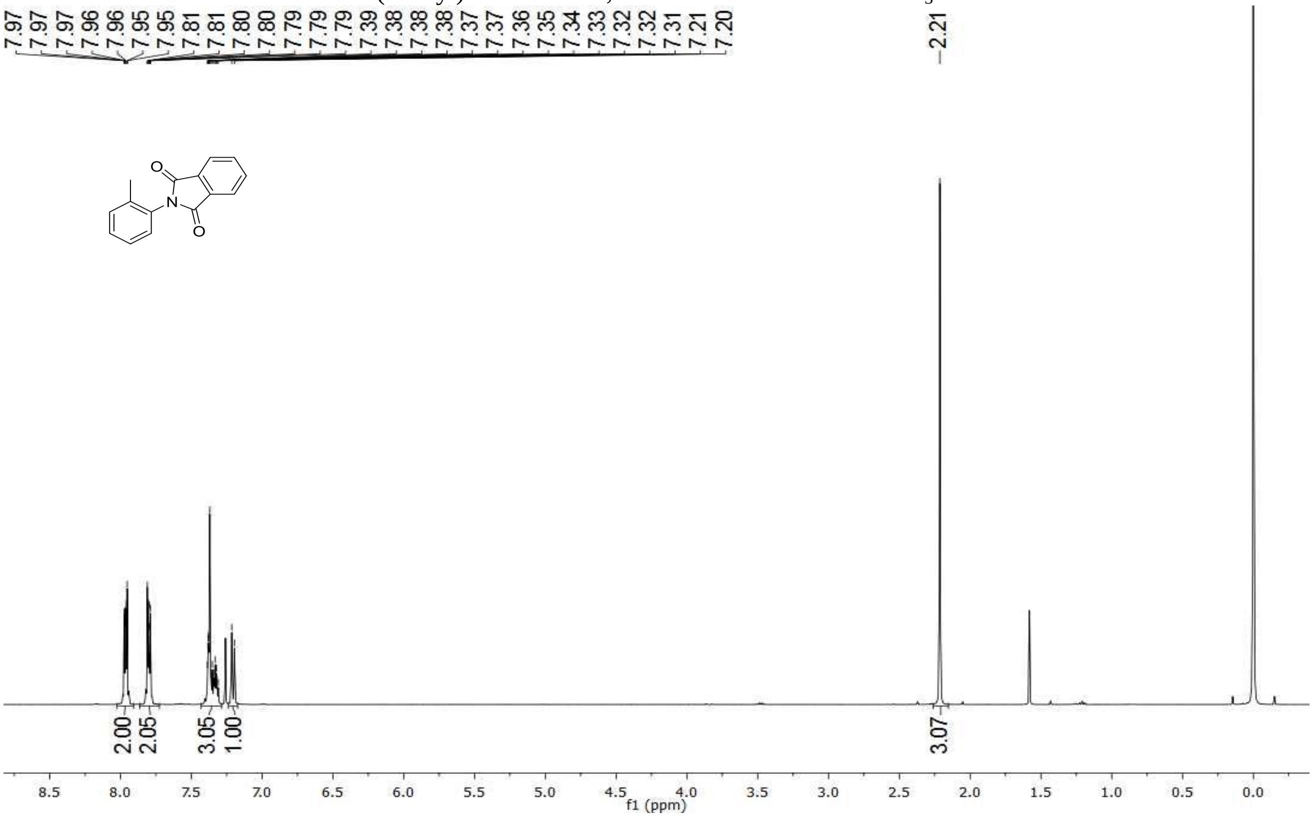

^{13}C NMR of 2-(o-tolyl)isoindoline-1,3-dione at 101 MHz in $CDCl_3$ at 298 K

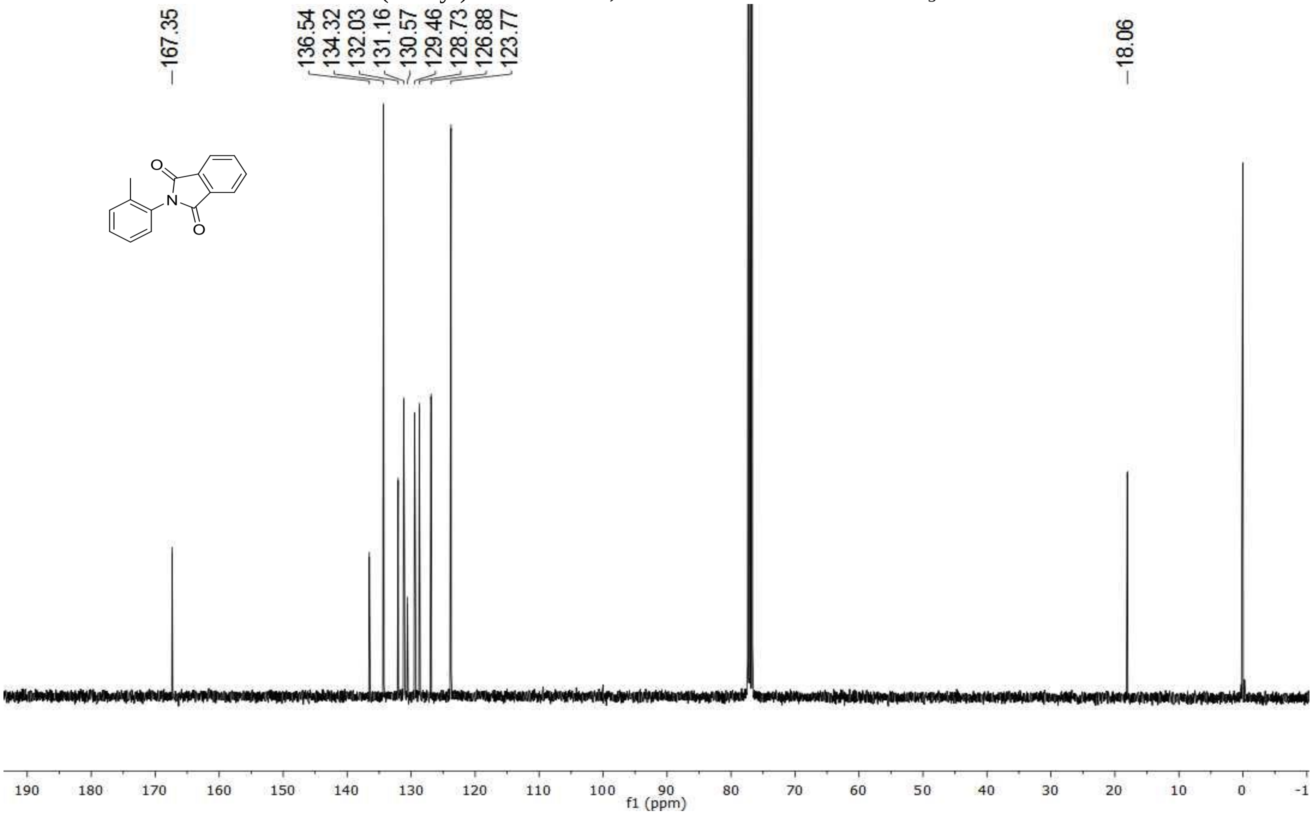

^{1}H NMR of 2-(4-fluoro-3-(trifluoromethyl)phenyl)isoindoline-1,3-dione at 400 MHz in $CDCl_3$ at 298 K

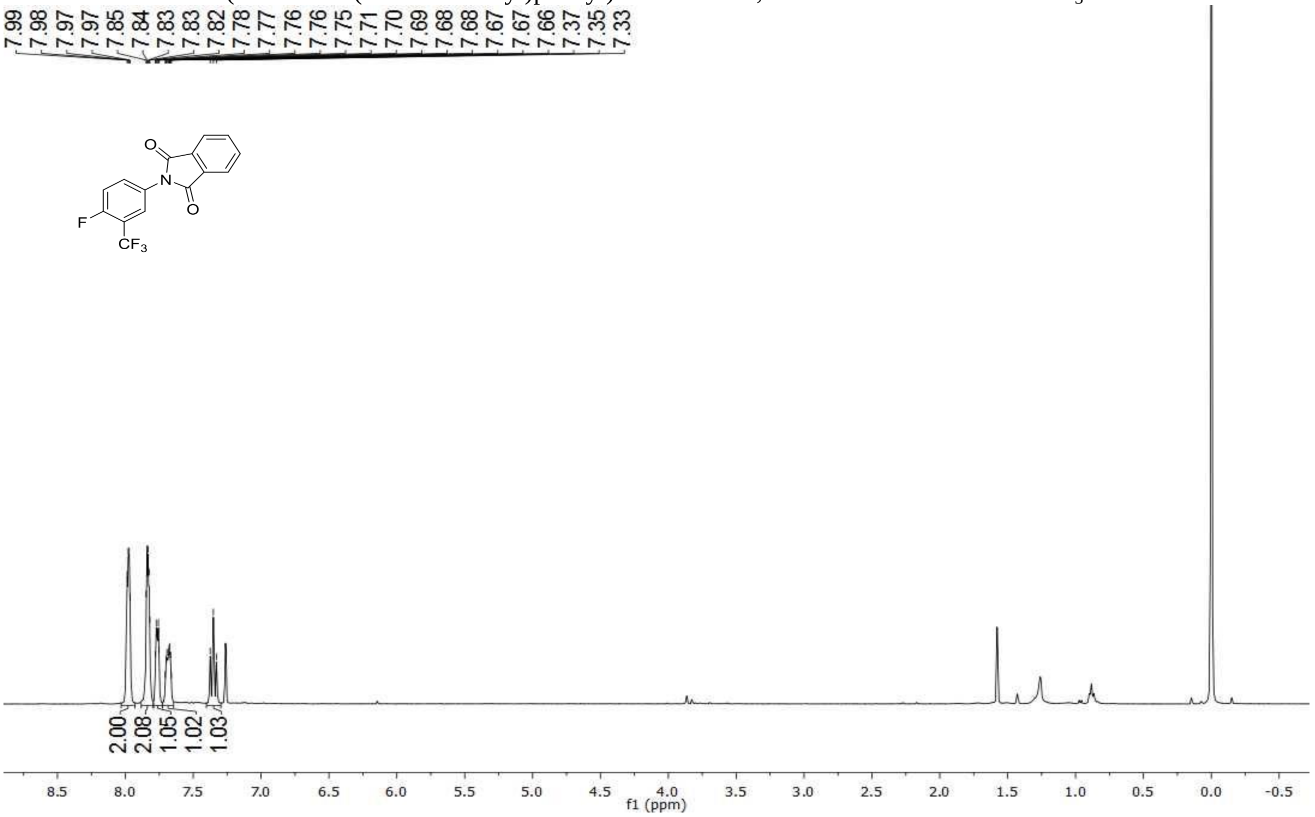

^{13}C NMR of 2-(4-fluoro-3-(trifluoromethyl)phenyl)isoindoline-1,3-dione at 101 MHz in $CDCl_3$ at 298 K

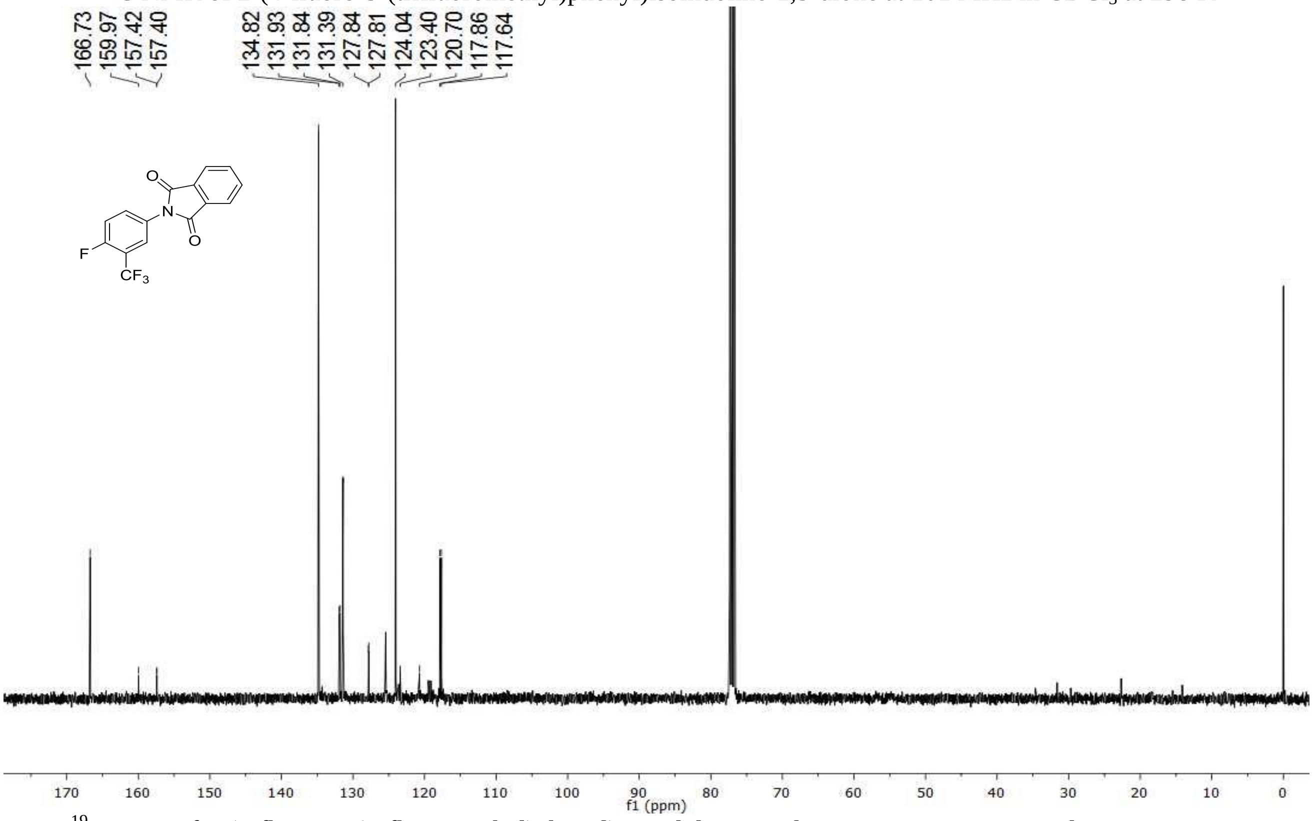

^{19}F NMR of 2-(4-fluoro-3-(trifluoromethyl)phenyl)isoindoline-1,3-dione at 376 MHz in $CDCl_3$ at 298 K

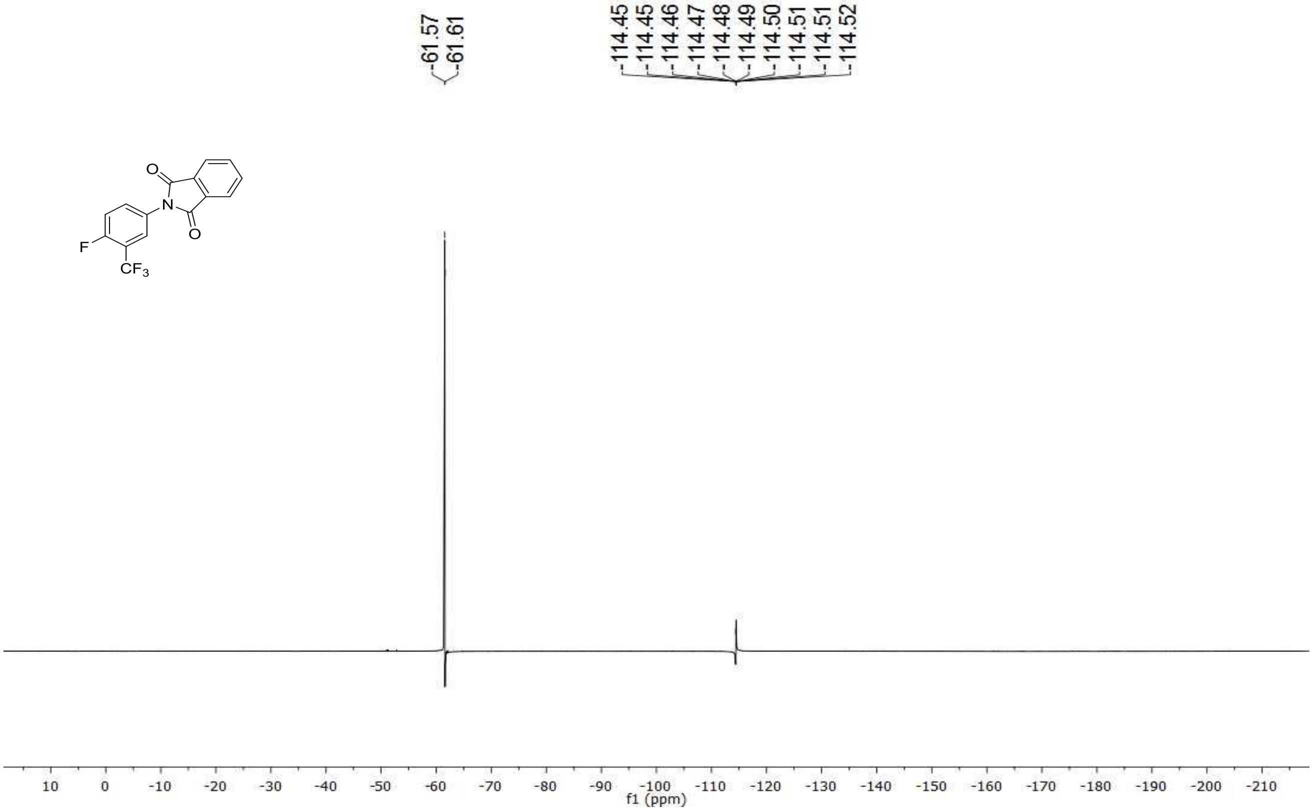

^{1}H NMR of 2-(2-chloro-4-nitrophenyl)isoindoline-1,3-dione at 400 MHz in $CDCl_3$ at 298 K

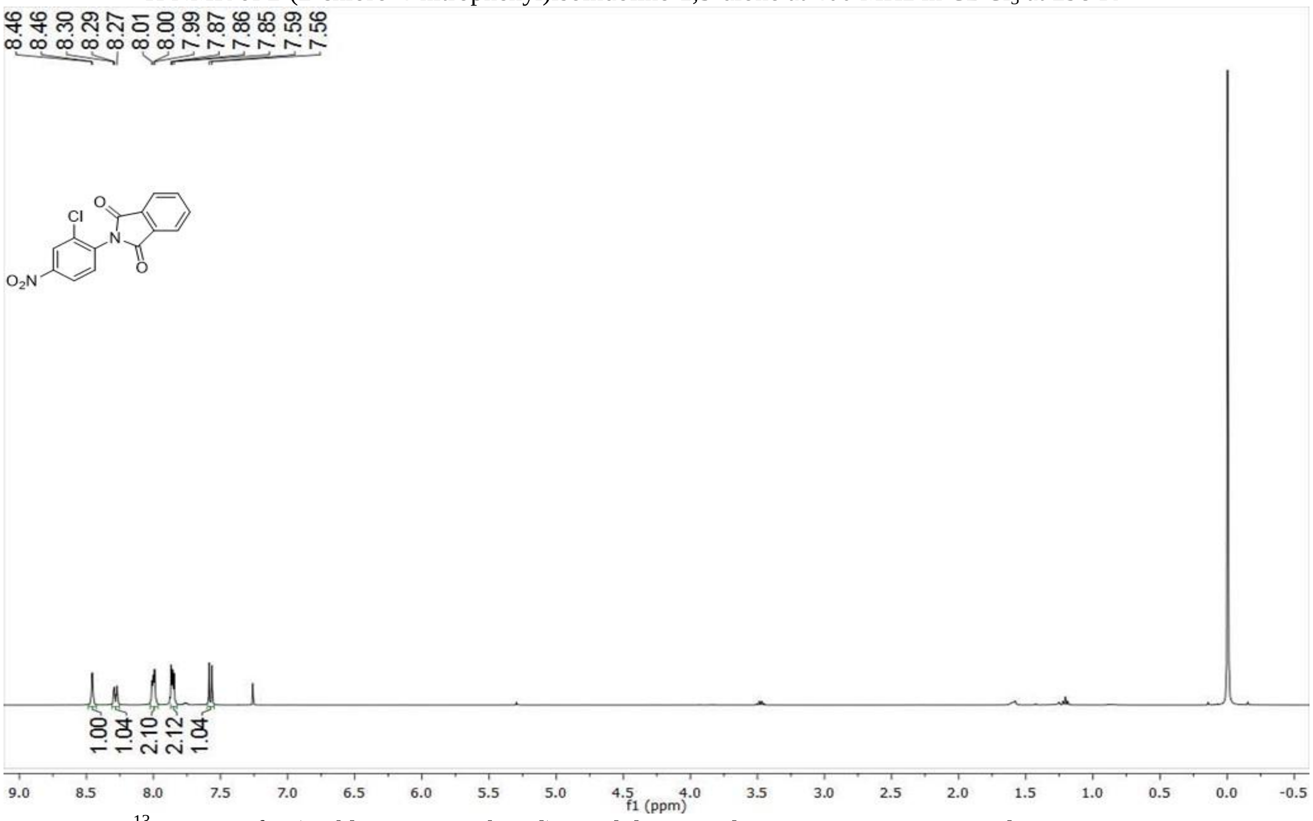

^{13}C NMR of 2-(2-chloro-4-nitrophenyl)isoindoline-1,3-dione at 101 MHz in $CDCl_3$ at 298 K

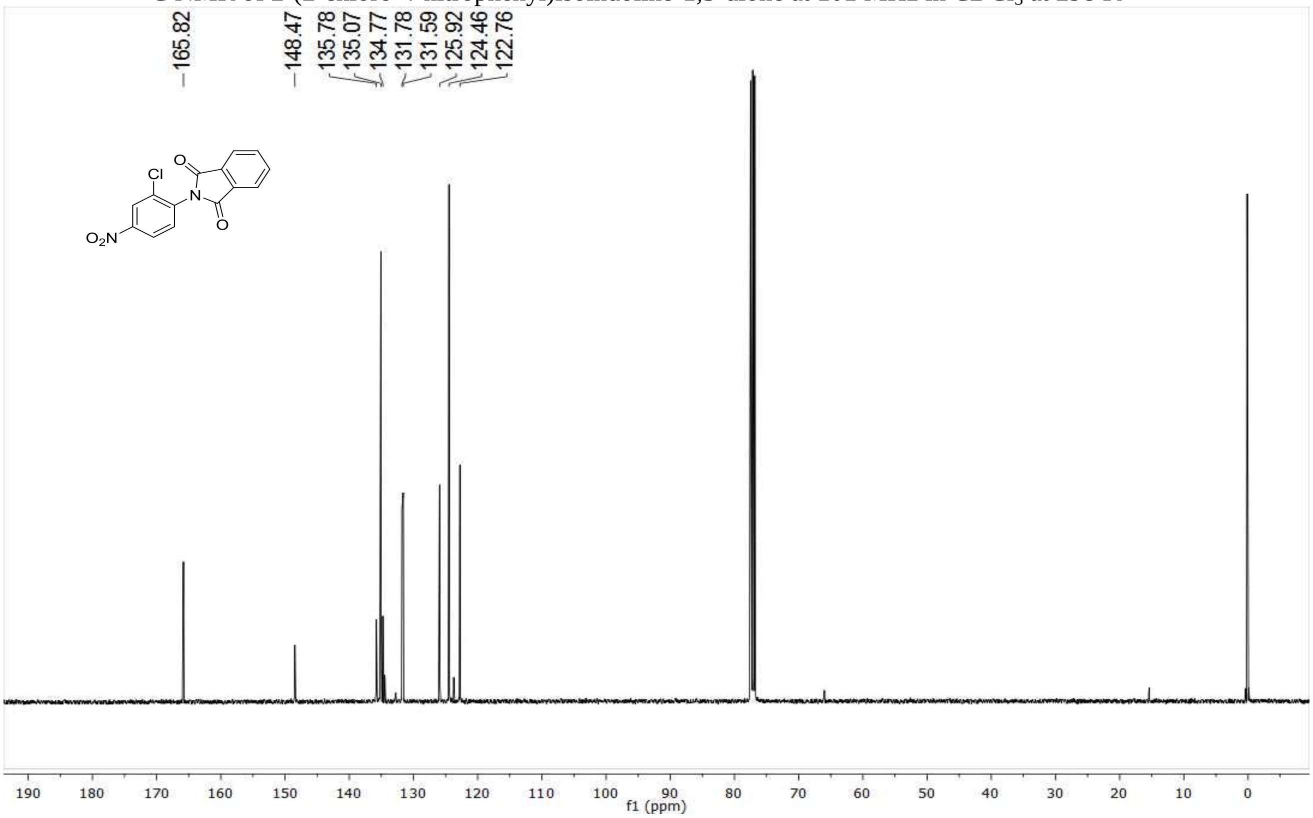

^{1}H NMR of methyl 4-aminobenzoate at 400 MHz in $CDCl_3$ at 298 K

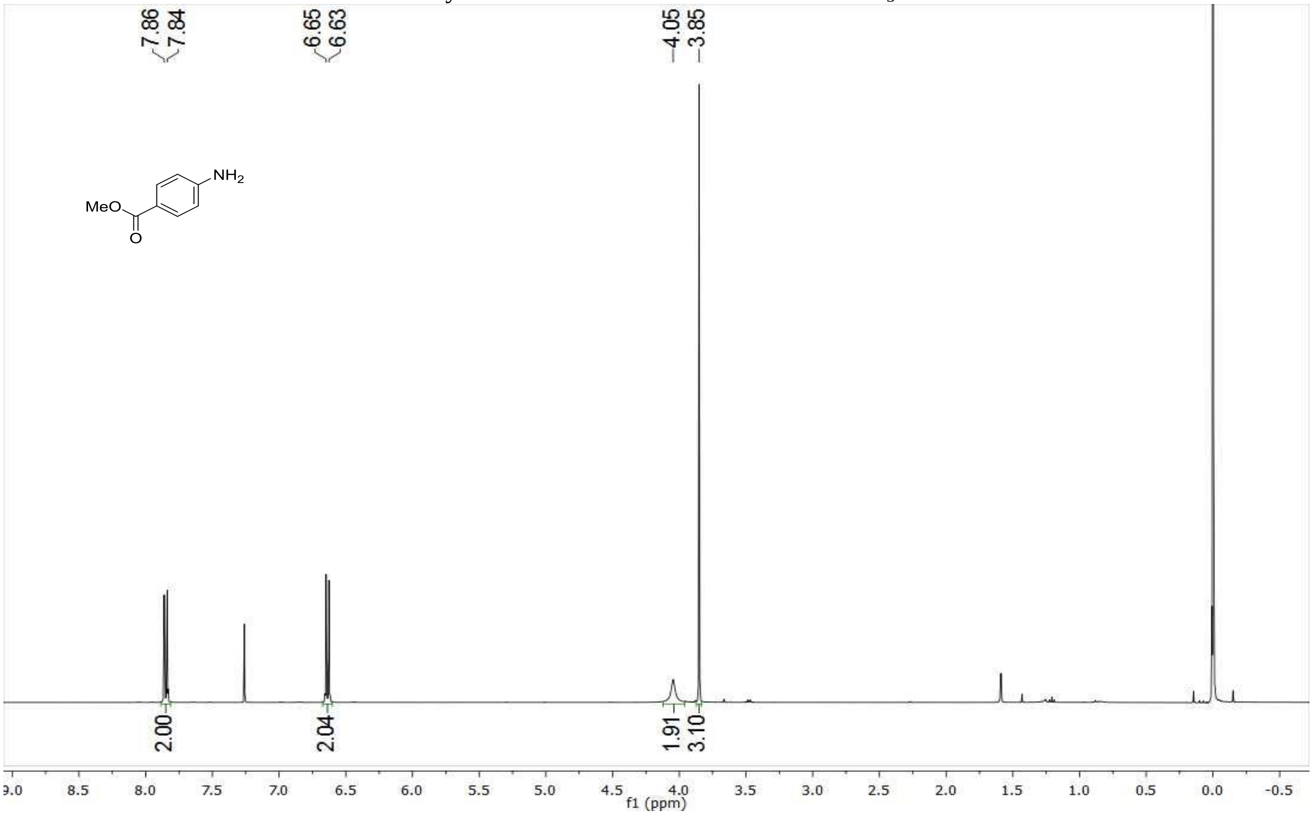

^{13}C NMR of methyl 4-aminobenzoate at 101 MHz in $CDCl_3$ at 298 K

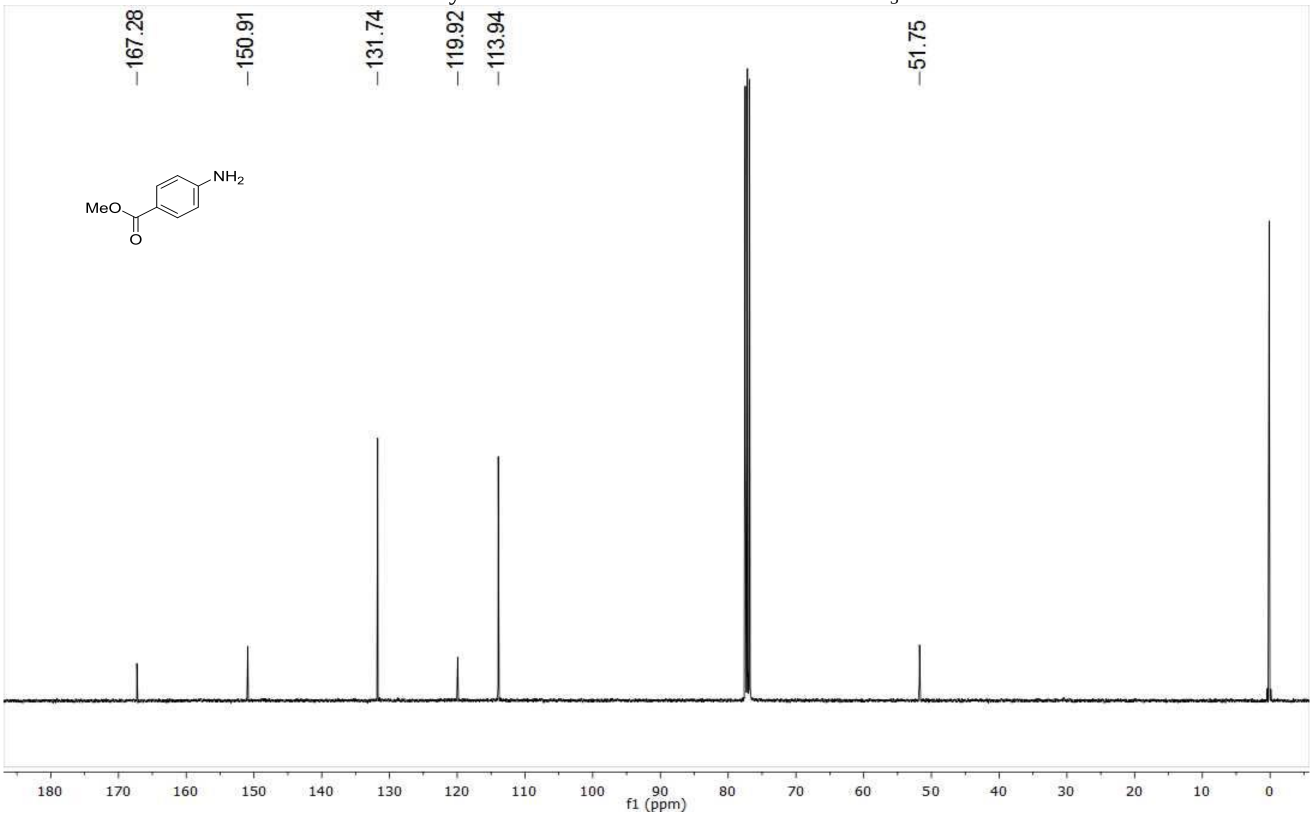

[1] (a) Carreras, V.; Sandtorv, A. H.; Stuart, D. R. *J. Org. Chem.*, **2017**, *82*, 1279-1284. (b) Seidl, T. L.; Sundalam, S. K.; McCullough, B.; Stuart, D. R. *J. org. Chem.*, **2016**, *81*, 1998-2009.

[2] Kim, H.; Kim, T.; Lee, D. G.; Roh, S. W.; Lee, C. *Chem. Commun.*, **2014**, *50*, 9273-9276

[3] Yuan, Y. C.; Kamaraj, R.; Bruneau, C.; Labasque, T.; Roisnel, T.; Gramage-Doria, R. *Org. Lett.*, **2017**, *19*, 6404-6407

[4] Hügel, H. M.; Rix, C. J.; Fleck, K. *Synlett*,, **2006**, *14*, 2290-2292

[5] Allen, L. J.; Cabrera, P. J.; Lee, M.; Sanford, M. S. *J. Am. Chem. Soc.*, **2014**, *136*, 5607-5610

[6] Ji, F.; Li, J.; Li, X.; Guo, W.; Wu, W.; Jiang, H. *J. Org. Chem.*, **2018**, *83*, 104-112

[7] Garcia-Barrantes, P. M.; Cho, H. P.; Niswender, C. M.; Byers, F. W.; Locuson, C. W.; Blobaum, A. L.; Xiang, Z.; Rook, J. M.; Conn, P. J.; Lindsley, C. W. *J. Med. Chem.*, **2015**, *58*, 7959-7971

[8] Fountoulaki, S.; Daikopoulou, V.; Gkizis, P. L.; Tamiolakis, I.: Armatas, G. S.: Lykakis, I. N. *ACS Catal*. **2014**, *4*, 3504-3511
